# Structural characterisation of a cysteine-rich conotoxin, sigma(σ)S-GVIIIA, extracted from the defensive venom of the marine cone snail *Conus geographus*

**DOI:** 10.1042/BCJ20240753

**Published:** 2025-05-23

**Authors:** Yoshimi Peck, David T. Wilson, Danica Lennox-Bulow, Julien Giribaldi, Jamie Seymour, Sebastien Dutertre, K. Johan Rosengren, Michael J. Liddell, Norelle L. Daly

**Affiliations:** 1 Australian Institute of Tropical Health and Medicine, James Cook University, Cairns, Queensland, Australia; 2 IBMM, University of Montpellier, CNRS, ENSCM, Montpellier, France; 3 School of Biomedical Sciences, The University of Queensland, Brisbane, Queensland, Australia; 4 College of Science and Engineering, James Cook University, Cairns, Queensland, Australia

**Keywords:** alphafold, conotoxin, cysteine-rich peptide, growth factor cystine knot (GFCK), nuclear magnetic resonance (NMR) spectroscopy

## Abstract

The activity of the serotonin type 3 (5-HT_3_) receptor is associated with neurodegenerative, inflammatory and metabolic diseases, neuropsychiatric disorders and cancer. Structural analysis of modulators of this receptor is likely to aid in future medicinal chemistry studies aimed at developing lead molecules targeting this receptor. Here, we report the structure of a cone snail venom peptide that was purified from the crude venom of *Conus geographus* and shown to be an antagonist of the 5-HT_3_ receptor more than 25 years ago, sigma(σ)S-GVIIIA. This lag in structural characterisation studies is likely due to challenges in isolating the native peptide and difficulties in producing synthetic peptide due to the presence of ten cysteine residues involved in five disulfide bonds. Using NMR spectroscopy, we show that σS-GVIIIA adopts a growth factor cystine knot (GFCK) fold. This is the first example of a cone snail venom peptide experimentally determined to contain the GFCK structural motif and the first example of a 5-HT_3_ receptor antagonist containing this motif. Our study also highlights complexities in the use of artificial intelligence (AI)-based structure prediction models. Peptide structure predictions using AlphaFold 3 were consistent with our NMR structure when the input sequence contained the well-conserved precursor sequence but inconsistent when the precursor sequence was excluded. AI-based structure prediction of proteins is a rapidly advancing field, but this inconsistency emphasises the need for more experimental structural training data when novel structures are involved, as was the case here for a cysteine-rich peptide.

## Introduction

Cone snails (*Conus* spp.) are predatory marine gastropods found predominantly in tropical or sub-tropical regions [1]. Despite their slow movement, they can immobilise prey and predators quickly by injecting a potent venom [2]. The venom has been found to be a cocktail of pharmacologically active molecules dominated by cysteine-rich peptides referred to as conotoxins [3].

Conotoxins are predominantly neurotoxins that act on ion channels and membrane receptors in the nervous system [4]. They generally have well-defined structures braced by disulfide bonds [3], which confer enhanced chemical and enzymatic stability [5–7]. Conotoxins are also generally potent and specific ligands to their target receptor [8], making them useful pharmacological tools and attractive drug leads for a range of applications including the treatment of pain, neuropsychiatric conditions, cardiac diseases and diabetes [9,10].

Although many conotoxins have been structurally and functionally characterised, they still represent only a small proportion of the more than 80,000 conotoxins estimated to be present in the more than 700 known *Conus* species [11–13]. Characterisation of novel conotoxins can be challenging for several reasons including structural complexity and low abundance in venom samples. Solid-phase peptide synthesis can be used to generate sufficient quantities of low-abundance peptides; however, for cysteine-rich conotoxins, the formation of the correct structural fold *in vitro* can be problematic without prior knowledge of the native disulfide pairing. For example, a conotoxin with ten cysteine residues can have 945 possible disulfide connectivities [14,15]. Thus, knowledge of the disulfide bond connectivities of cysteine-rich conotoxins is often required to allow the directed formation of the native disulfide bonds during *in vitro* synthesis.

σS-GVIIIA is one of the many conotoxins that has not been well characterised, particularly in terms of structure. It has only been found in the defensive venom of *Conus geographus* and consists of 41 amino acids and contains multiple post-translational modifications, including a bromotryptophan, hydroxy-proline, C-terminal amidation and five-disulfide bonds [16]. It is classified into the S-gene superfamily, has a type VIII cysteine framework and its competitive antagonism at the serotonin type 3 (5HT_3_) receptor makes it the first, and to our knowledge, currently the only member of the σ pharmacological family.

The 5-HT_3_ receptor has been recognized as a clinically important target because its activity has been implicated in substance addiction, clinical depression, cancer, diabetes and inflammatory and neurodegenerative diseases [17–20]. The low abundance of σS-GVIIIA in the venom and highly cysteine-rich nature has made it challenging to work with and has contributed to the limited follow-up studies after the initial identification of the pharmacological activity of this peptide over two decades ago [16]. Consequently, there have been no structural studies, and the disulfide connectivity has not been determined.

In the current study, we have isolated native σS-GVIIIA from milked defensive venom to identify the native three-dimensional (3D) fold and cysteine connectivities using nuclear magnetic resonance (NMR) spectroscopy. We found that σS-GVIIIA is the first conotoxin to contain the growth factor cystine knot (GFCK) motif. The GFCK is a structural motif initially identified in multiple growth factor proteins approximately three decades ago [21]. No structural studies for invertebrate venom peptides containing the GFCK motif have been reported to our knowledge, in contrast with the common inhibitor cystine knot (ICK) [22,23]. σS-GVIIIA is also the first example of a 5-HT_3_ receptor antagonist containing the GFCK motif.

To explore the reliability of modern artificial intelligence (AI)-based structure prediction models, we compared our experimental structure of σS-GVIIIA with the predicted structure from AlphaFold 3 and also predicted the structure of σS-GVIIIA in complex with the 5-HT_3_ receptor. The aim of these analyses was to provide insight into the strengths and limitations of AI predictions and potential insight into pharmacophore development for the 5-HT_3_ receptor.

## Results

### The molecular profile of *C. geographus* venom in response to offensive and defensive stimuli

Crude *C. geographus* venom was collected from multiple live specimens through defence and predation-provoked stimuli. The liquid chromatography–mass spectrometry (LC–MS) profile of each milking was obtained immediately following each venom collection, and significant variation was observed. The relative abundance of σS-GVIIIA in the crude venom fluctuated between individuals and between subsequent milkings of the same individual. To illustrate this, a comparison of the LC–MS chromatograms of crude defensive venom from two individual *C. geographus* specimens (A and B) milked on the same day is shown in Supplementary Figure S1A and B. These profiles show considerable differences, and σS-GVIIIA was generally not detected in venom samples from specimen A but was detected in the venom of specimen B. An uncharacterized 8916 Da peptide and I_3_-GXIA were the most abundant components in the venom of specimen A, while μ-GIIIA conotoxin was the major component in the venom of specimen B. The relative intensity of σS-GVIIIA in specimen B also varied in subsequent milkings taken on different days (Supplementary Figure S1B and C).

Variation in venom composition between offensive and defensive venom was also observed for the venom from *C. geographus* specimen C, and σS-GVIIIA was not detected in the offensive venom but only detected in the defensive venom samples (Supplementary Figure S1D). Analysis of LC–MS profiles showed the only common mass found in both offensive and defensive venom was a 3036 Da peptide, which is predicted to be ω-GVIA.

### Purification of the σS-GVIIIA conotoxin from crude venom

To collect σS-GVIIIA for NMR experiments (~0.6 mg), milkings were carried out with defensive stimuli over 36 months. The collected venom was pooled and fractionated using reversed-phase high-performance liquid chromatography/mass spectrometry (RP-HPLC/MS) (Supplementary Figure S2). The isolated peptide was identified as σS-GVIIIA (sequence ID P58924) based on the mass of 4188/4192 Da (Supplementary Figure S3) and NMR spectroscopy analysis. The presence of two molecular masses is due to the presence of the bromotryptophan (residue 34), which has two naturally occurring isotopes, Br^79^ and Br^81^, at a 51:49 ratio.

### Disulfide bond prediction based on the preliminary NMR structure of σS-GVIIIA

Purified native σS-GVIIIA was solubilised in 90% H_2_O:10% D_2_O (v/v) at a final concentration of approximately 0.26 mM. Two-dimensional spectra, including ^1^H–^1^H TOCSY (total correlation spectroscopy), ^1^H–^1^H NOESY (nuclear Overhauser effect spectroscopy), ^1^H–^1^H DQF-COSY (double-quantum filtered correlation spectroscopy) and ^1^H–^13^C HSQC (heteronuclear single quantum coherence spectroscopy), were collected for subsequent sequence-specific assignments and structure calculations [24,25]. The signal-to-noise ratio of the ^1^H–^15^N HSQC spectrum was low due to the low concentration of the sample, and the data were not useful in the structure calculations.

**Figure 1:**
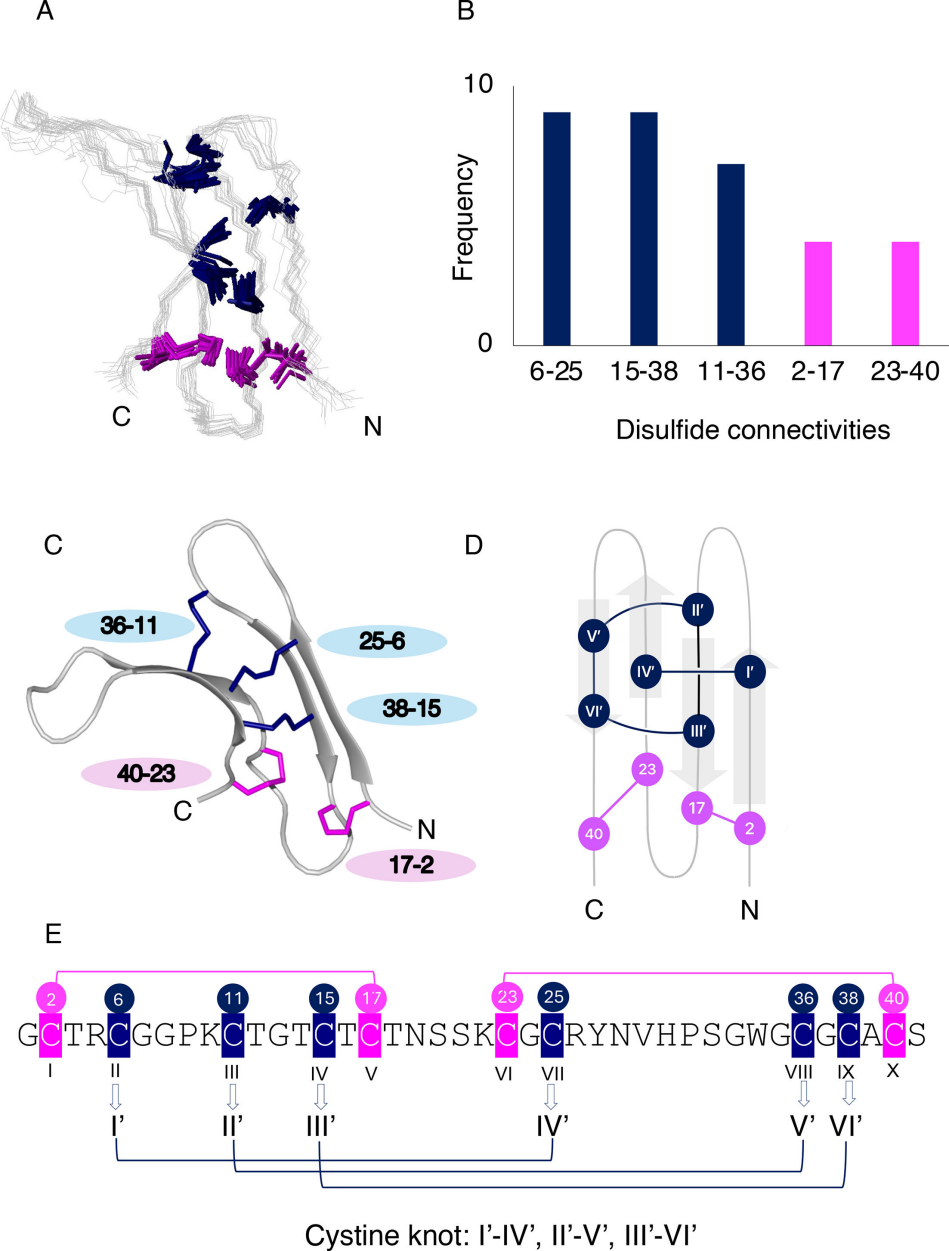
Determination of the structure of σS-GVIIIA. (**A**) The initial NMR structures were calculated without disulfide bond restraints. Cysteine side chains are highlighted in navy or pink. The ensemble of 20 structures with the lowest target functions is shown. (**B**) The inter-sulfur distance analysis of the preliminary structures of σS-GVIIIA. The number of structures, out of the 20 lowest energy structures, which have particular sulfur–sulfur distances less than 3.0 Å, are plotted. (**C**) The final structure of σS-GVIIIA (PDB:9EBE). The structure with the lowest target function is shown. (**D**) The schematic figure of σS-GVIIIA. The knot-forming cysteine residues are shown in navy, whereas cysteine residues not involved in the knot are shown in magenta. (**E**) The sequence of σS-GVIIIA with re-numbered cysteine residues involved in the cystine knot. All ten cysteine residues were numbered as I–X, and then knot-forming six cysteine residues were re-numbered as I’–VI’ and highlighted in navy. The cysteine residues, not involved in the knot, are highlighted in magenta.

Initial NMR structures of σS-GVIIIA were calculated using CYANA (combined assignment and dynamics algorithm for NMR applications) [26,27] without any disulfide bond restraints. An ensemble of 20 structures corresponding to the lowest target function is shown in Figure 1A. These structures were relatively well-defined despite the lack of disulfide bond restraints (backbone root-mean-square deviation [RMSD] of 0.84 ± 0.40 Å for the well-defined region, residues 3–5, 13–15, 25–28 and 35–38, and 1.33 ± 0.67 Å for residues 1–41) and therefore could be used to predict the most likely disulfide bonds. The prediction was based on the distance between sulfur atoms in pairs of cysteine residues. The mean, standard deviation, minimum and maximum distance of each cysteine pair are summarised in Supplementary Table S1, and the specific distances in the individual structures are shown in Supplementary Figure S4. The five cysteine pairs with the lowest mean distance corresponded to Cys^2^-Cys^17^, Cys^6^-Cys^25^, Cys^11^-Cys^36^, Cys^15^-Cys^38^ and Cys^23^-Cys^40^, indicating that this is the most likely connectivity. Analysis of the cysteine residues that are not located in close proximity can also be informative for predicting the disulfide connectivity, and in this case, the most definitive finding from the analysis indicated that Cys^2^ is only in close proximity to Cys^17^ and does not have any other option to pair based on the individual distances (Supplementary Figure S4).

Analysis of the sulfur–sulfur distances has been done in previous studies of disulfide-rich peptides using cut-off distances [28,29]. We used a cut-off distance of 3.0 Å between the sulfur atoms based on our initial analysis of the average distances. This analysis indicated that the most likely connectivity for σS-GVIIIA is Cys^2^-Cys^17^, Cys^6^-Cys^25^, Cys^11^-Cys^36^, Cys^15^-Cys^38^ and Cys^23^-Cys^40^ (Figure 1B).

### Description of the overall structure and discovery of the GFCK motif in σS-GVIIIA

The final structures for σS-GVIIIA (the PDB and BMRB codes are 9EBE and 31213, respectively) were calculated with disulfide bond restraints based on the most likely connectivities predicted by the inter-sulfur distance analysis. The NMR-derived structure with the lowest target function is shown in Figure 1C, and the structure ensemble containing the 20 structures with the lowest target function is shown in Supplementary Figure S5. The structural statistics are listed in Table 1. The main elements of the secondary structure in σS-GVIIIA are four anti-parallel β-strands, forming two β-hairpins. All four β-strands, referred to as β1–β4, are not found in the same plane, but the β-hairpins are facing each other (Figure 1C and D).

**Table 1:** Statistics and analysis of the σS-GVIIIA conotoxin structure.

**Experimental restraints**	
Interproton distance restraints^1^	
Intraresidue	170
Sequential	98
Medium range (i-j < 5)	13
Long range (i-j ≥ 5)	65
Total	346
Dihedral-angle restraints^2^	50
**Restraint statistics**	
Average number of violations per structure^1^	
NOE restraints > 0.2 Å	0
Dihedral restraints > 2°	0
**Restraint violations**	
Distance restraints (Å)^1^	
RMS deviations	0.0123 ± 0.0009
Dihedral-angle restraints (°)^1^	
RMS deviations	0.069 ± 0.050
**Structural quality**	
RMSD from average structure (Residues 1–41) (Å)^3^	
Backbone atoms	0.68 ± 0.138
All heavy atoms	1.15 ± 0.30
RMSD from average structure (selected residues)^5^ (Å)^3^	
Backbone atoms	0.46 ± 0.28
All heavy atoms	1.10 ± 0.25
Ramachandran statistics (%)^4^	
Most favoured regions	73.0
Additionally allowed regions	27.0
Generously allowed regions	0
Disallowed regions	0
Global quality scores (raw/Z scores)^4^	
Verify3D	0.16/−4.82
Prosall (−ve)	0.66/0.04
PROCHECK (all)	−0.89/−5.26
MolProbity clashscore	8.87 / 0.00

1 Derived from CYANA [26].

2 Predicted from TALOS-N [30,31].

3 Calculated using MOLMOL [32].

4 Evaluated by PSVS [33].

5 Residues 3-6, 13-16, 25-28 and 35-38.

Analysis of the topology of the disulfide bonds in σS-GVIIIA indicated the presence of a cystine knot motif at the core (Cys^6^-Cys^25^, Cys^11^-Cys^36^ and Cys^15^-Cys^38^ with two additional disulfide bonds (Cys^2^-Cys^17^ and Cys^23^-Cys^40^ located away from the core of the peptide; Figure 1C and D). For comparative purposes, we will refer to the disulfide bonds directly involved with the cystine knot as Cys^I’^-Cys^IV’^, Cys ^II’^-Cys^V’^ and Cys^III’^-Cys^VI’^, respectively (Figure 1E). In this Roman numeral numbering system, the cysteine residues that do not contribute directly to the knot motif have been excluded. The additional disulfide bonds will still be described as Cys^2^-Cys^17^ and Cys^23^-Cys^40^, without using Roman numerals.

**Figure 2:**
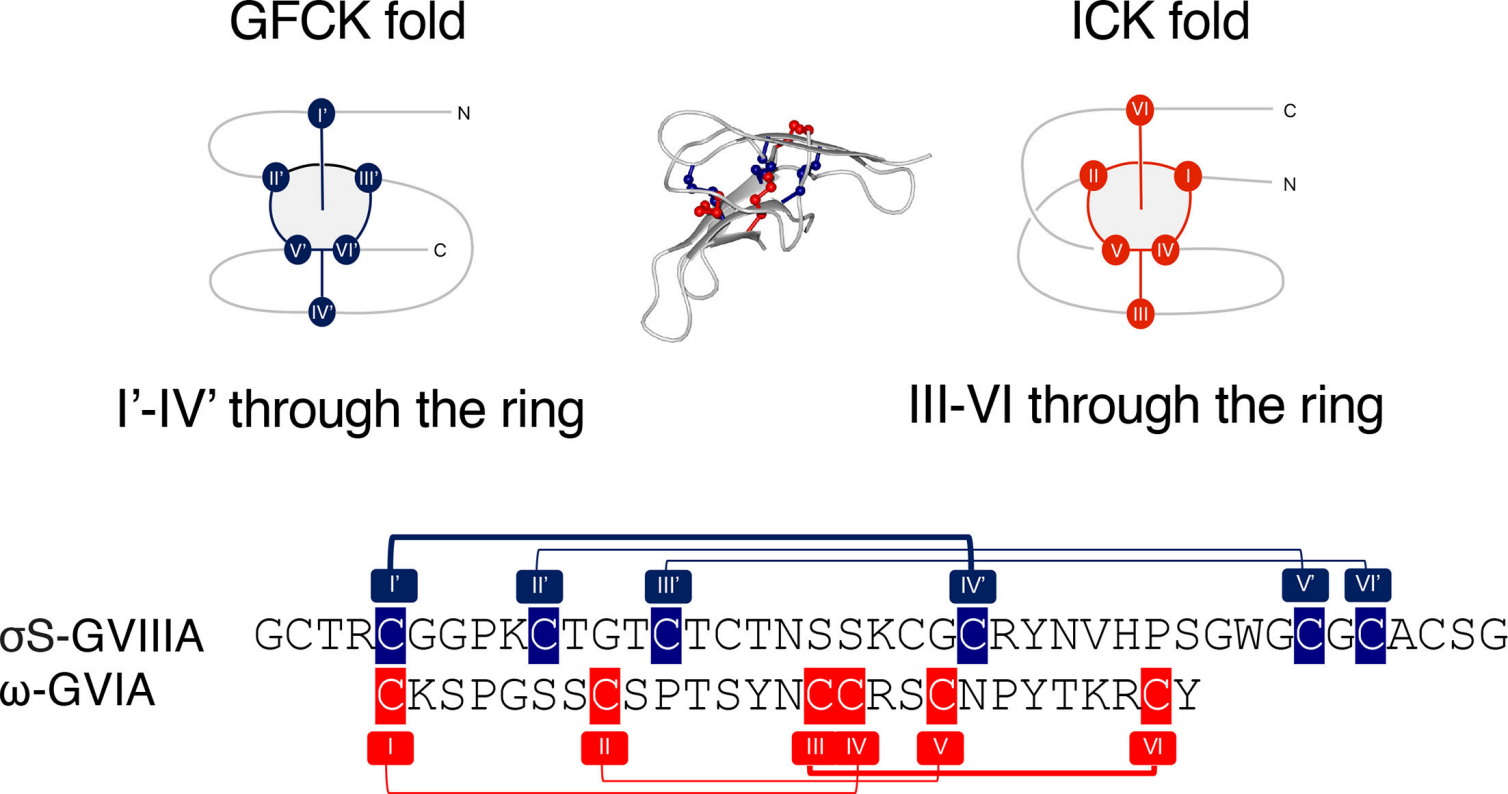
Comparison of the GFCK and ICK motifs. The GFCK motif is shown in navy blue in σS-GVIIIA (PDB:9EBE), and the ICK motif is shown in red in ω-GVIA (PDB:2CCO) for both the schematic representations of the knot structures and the sequences. Cysteine residues involved in the knot are numbered using Roman numerals, I–VI for ICK and I’–VI’ for GFCK. Cysteine residues, which are not involved in the knot, have not been highlighted. GFCK, growth factor cystine knot.

**Figure 3:**
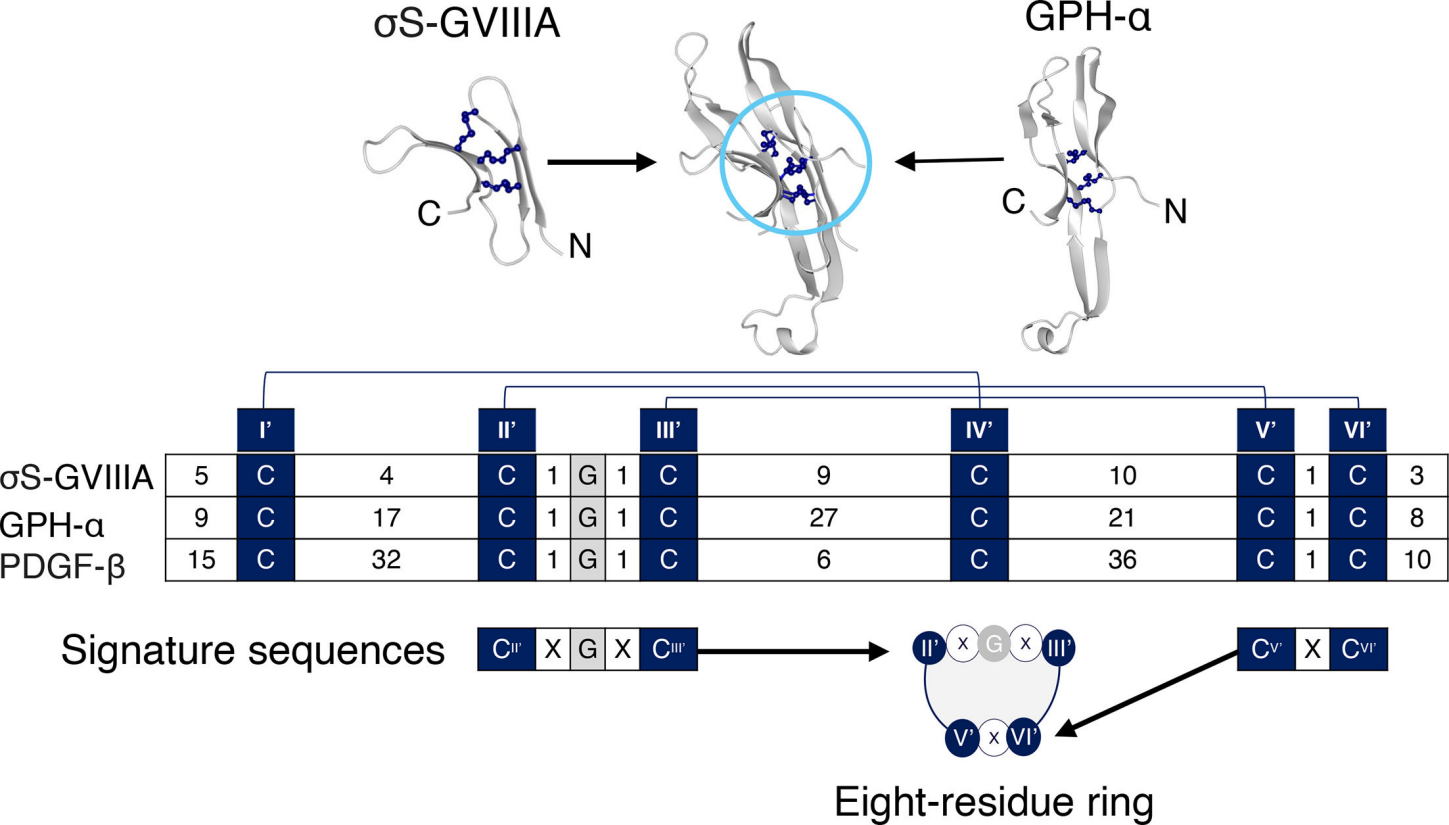
Comparison of the GFCK motif present in σS-GVIIIA and GPH-α. The disulfide bonds involved in the GFCK motif in the structures of GPH-α (PDB:1HPR) and σS-GVIIIA (PDB:9EBE) are shown in navy blue and superimposed in the middle of the diagram. The signature sequences of the eight-residue ring of the GFCK motif, along with the number of residues in the inter-cysteine loops for σS-GVIIIA, GPH-α and PDGF-β, and the disulfide bonds are shown in the lower half of the diagram. Cysteine residues, which are not participating in the knot formation, have not been highlighted. GFCK, growth factor cystine knot; GPH-α, glycoprotein hormone α-subunit; PDGF, platelet-derived growth factor β-subunit.

The σS-GVIIIA cystine knot was compared with the ICK motif [22], which is commonly found in conotoxins and has the same disulfide connectivity Cys^I^-Cys^IV^, Cys^II^-Cys^V^ and Cys^III^-Cys^VI^ in the knot (Figure 2). The knot motif of σS-GVIIIA was not superimposable with an ICK motif containing peptide, namely, conotoxin ω-GVIA (PDB: 2CCO), also originally isolated from *C. geographus* (Figure 2). Schematic figures of ω-GVIA and σS-GVIIIA highlight the major differences, where the Cys ^I’^-Cys^IV’^ bond threads through the ring of σS-GVIIIA, but Cys ^III^-Cys^VI^ threads through the ring in ω-GVIA (Figure 2). In contrast, the 3D topology of the knot motif was found to be superimposable with the GFCK motif of the human glycoprotein hormone α subunit (GPH-α) (PDB: 1HRP, Figure 3).

The signature sequence patterns of the GFCK motif have been established previously as CXGXC and CXC, where X refers to any single residue, and collectively these eight residues, including two disulfide bonds, form a ring as a part of the GFCK motif [34]. σS-GVIIIA contains the GFCK motif signature sequence and the eight-residue ring (Figure 3), including the presence of glycine in the middle, which is likely to prevent the steric clashes with the Cys^I’^-Cys^IV’^ disulfide bond that penetrates through the ring [34,35].

The two additional intra-molecular disulfide bonds, not present in the knot motif of σS-GVIIIA, appear to be uncommon based on a search of GFCK-containing proteins in the Protein Data Bank. The most similar arrangement of disulfide bonds to σS-GVIIIA was found in the vertebrate specific hormone GPH-α (PDB:1HRP) [36] (Supplementary Figure S6).

### Potential GFCK-containing peptides in *Conus* venom

**Figure 4:**
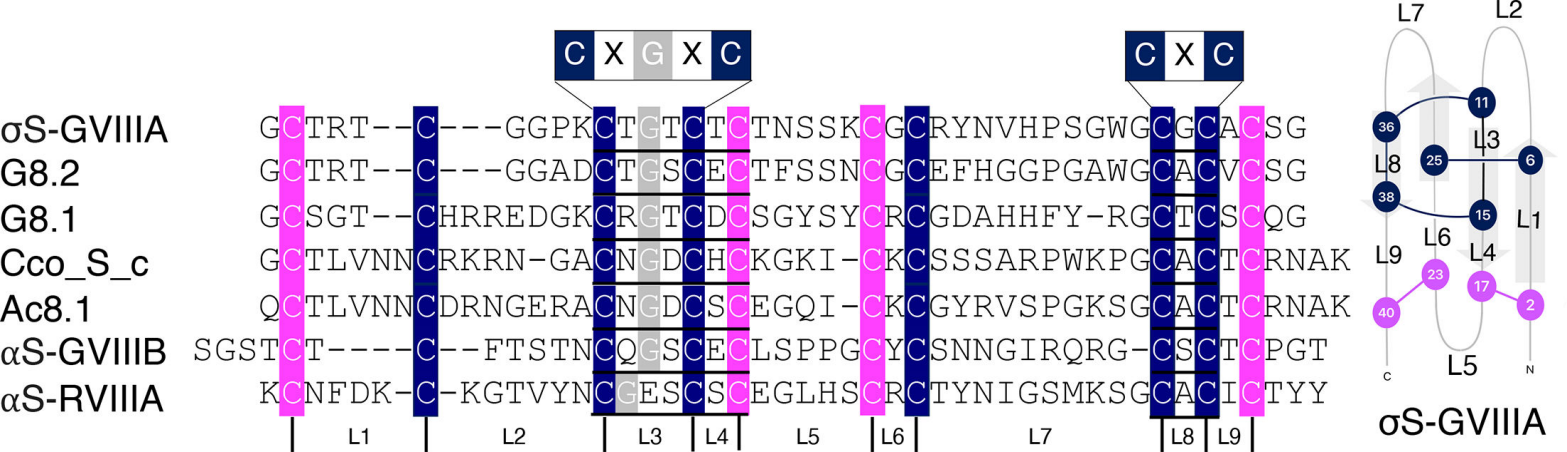
Sequence alignment of selected class VIII cysteine framework peptides. The GFCK signature sequences are indicated at the top of the sequence alignments. The cysteines involved in the knot formation are shown in navy while cysteine residues not participating in the knot formation are shown in magenta. The loop regions between cysteine residues are indicated as L1-L9 under the sequence as well as in the schematic figure of the σS-GVIIIA structure. GFCK, growth factor cystine knot.

The ConoServer database [37] of *Conus* venom peptides was searched for peptides containing the signature sequences of the GFCK motif (CXGXC and CXC). This sequence motif was found in the majority of peptides with the type VIII cysteine framework. Alignment of all peptides containing the type VIII cysteine framework showed 31 out of 36 sequences contained the GFCK motif signature sequences found in σS-GVIIIA. Cysteine residues not involved in the knot motif were also highly conserved (selected examples are shown in Figure 4). Sequence variability is present at inter-cysteine loops 1, 2 ,5 and 7; in addition, αS-RVIIIA (*C. radiatus*) showed variability in loop 3, where the sequence is CGXXC instead of CXGXC.

### AlphaFold prediction of the 3D structure of σS-GVIIIA in isolation

**Figure 5:**
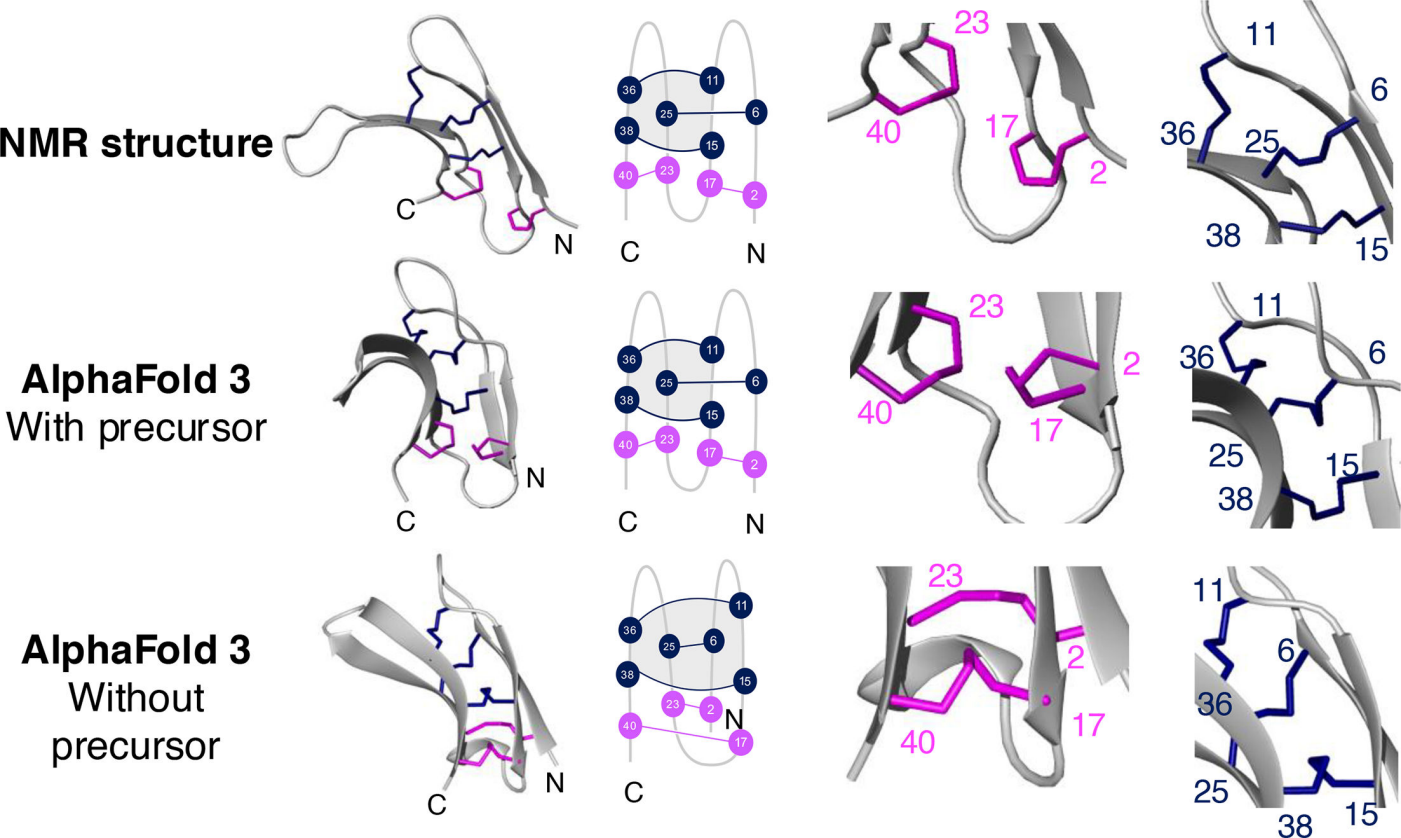
Comparison of the NMR-derived structure of σS-GVIIIA with AlphaFold 3 predictions with or without precursors. A ribbon and schematic representation of the three-dimensional structures is shown. The precursor region (for the middle structure) is not shown. The NMR-derived structure of σS-GVIIIA represents the structure with the lowest target function. The zoomed-in regions highlight two bonds not involved in the cystine knot (shown in magenta) and the three bonds involved in the knot (shown in blue). The cysteine residues are numbered with Arabic numerals. NMR, nuclear magnetic resonance.

Prediction of the structure of σS-GVIIIA with AlphaFold 3 [38] showed agreement with our NMR structure when the precursor sequence (signal peptide and proregion) was included in the input sequence (Figure 5). The backbone RMSD between the lowest energy NMR structure and the most likely prediction from AlphaFold 3 was 0.791 Å for the well-defined regions (residues 3–5, 15–17, 25–28 and 35–38) and 1.598 Å for residues 1–41.

This agreement between our NMR structure and the AlphaFold 3 predictions only occurred when the precursor sequence was included in the input sequence. When the precursor sequence was not included, the predicted structures were inconsistent with the NMR structure as shown in Figure 5. In the predictions without the precursor sequence, the position of the β3 and β4 strands was altered. Consequently, the knot motif was not topologically formed despite containing the Cys^6^-Cys^15^, Cys^11^-Cys^36^ and Cys^15^-Cys^38^ disulfide bonds. These disulfide bonds were also predicted when the precursor sequence was included, but the additional two disulfide bonds were predicted differently, with Cys^2^-Cys^17^ and Cys^23^-Cys^40^ predicted when the precursor sequence was included, and Cys^2^-Cys^23^ and Cys^17^-Cys^40^ predicted when the precursor sequence was excluded (Figure 5). The structure predicted with the precursor region did not show any interaction between the mature peptide and the precursor.

To further explore the differences in the predicted disulfide connectivity and experimentally derived connectivity of σS-GVIIIA, we calculated structures with the three possible disulfide connectivities for Cys^2^, Cys^23^, Cys^17^ and Cys^40^ (Supplementary Figure S7A–C: Set 1, Set 2 and Set 3) with the connectivities, Cys^6^-Cys^25^, Cys^11^-Cys^36^ and Cys^15^-Cys^38^ included as disulfide bond restraints. The comparison of the target function and restraint violations among the three sets of structures (Supplementary Figure S7A–C) indicated that Set 1 (i.e. Cys^2^-Cys^17^ and Cys^23^-Cys^40^) provided the most likely disulfide connectivities for these four cysteine residues, consistent with the prediction based on our initial analysis.

One of the five highest ranked predicted structures was not fully oxidised when the structures were predicted without the precursor sequence, whereas all five predictions that included the precursor sequence were consistent with a fully oxidised form (data not shown). All ten cysteine residues are involved in disulfide bonds based on the mass spectrometry results (Supplementary Figure S3) and a previous study [16]; thus, the partially oxidised structure is inconsistent with the experimental data.

The predicted structure of σS-GVIIIA is also available online at the AlphaFold Database [39]. The precursor region is included for this prediction, and all five disulfide connectivities are consistent with the NMR structure [40].

Overall, the inclusion of precursor residues in the input sequence influenced the structure prediction of σS-GVIIIA, and the resulting structure is consistent with the structure derived from the NMR studies.

### AlphaFold 3 prediction of a complex of σS-GVIIIA with the 5-HT_3_ receptor

**Figure 6:**
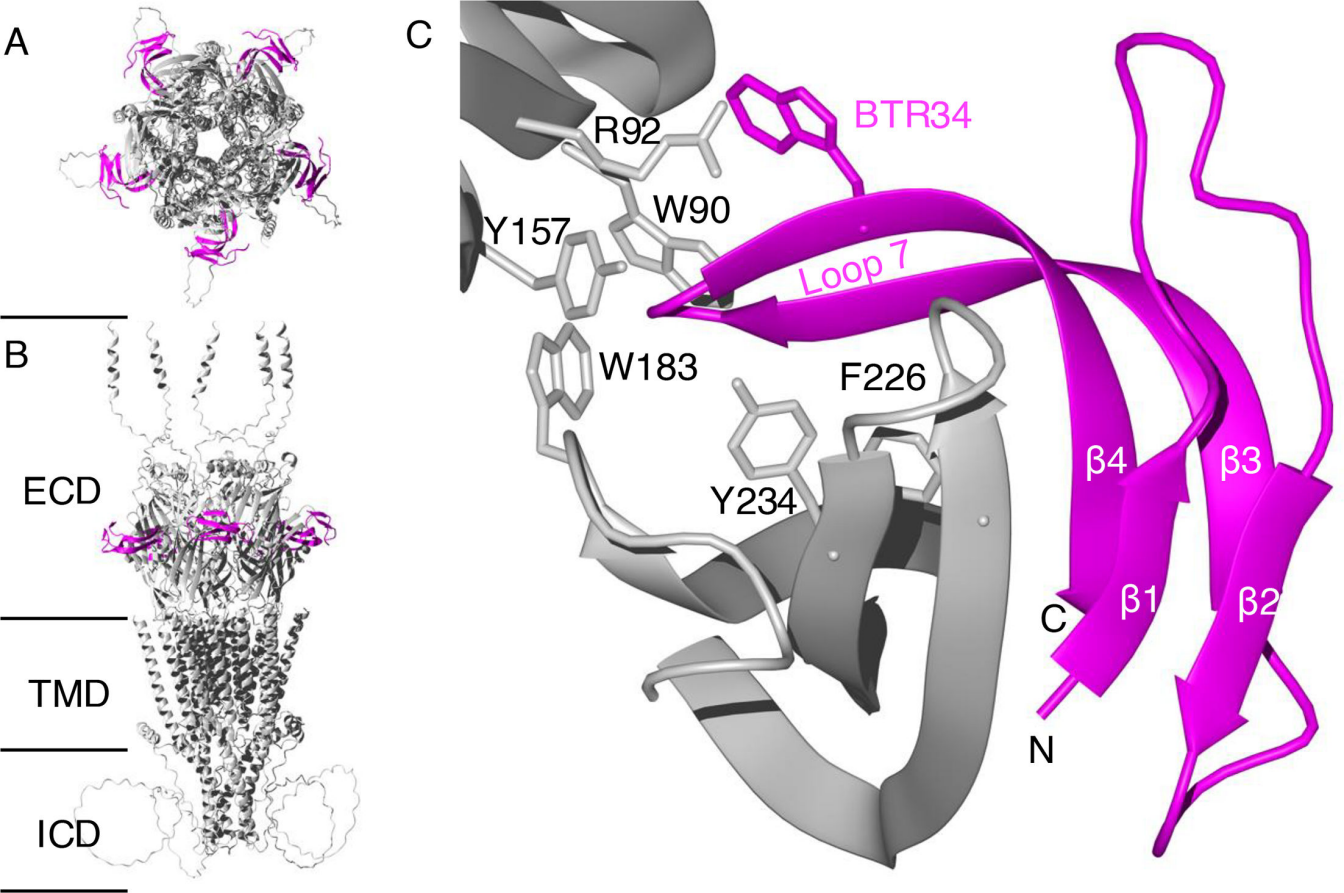
The ligand-receptor complex between σS-GVIIIA and the 5-HT_3_ receptor. (**A**) Side view of the σS-GVIIIA-5-HT_3_ receptor complex. (**B**) Top view of the σS-GVIIIA-5-HT_3_ receptor complex. The σS-GVIIIA interacting at the ECD of each subunit is shown in magenta. (**C**) The binding site of the σS-GVIIIA-5-HT_3_ receptor complex. The key residues on the orthosteric binding site on the 5-HT_3_ receptor are shown in silver stick format. The σS-GVIIIA conotoxin is shown in magenta. Bromotryptophan at residue 34 (BTR34) of σS-GVIIIA is shown in magenta stick format. The σS-GVIIIA consists of four β-strands, which are labelled as β1 to β4. ECD, extracellular domain; TMD, transmembrane domain; ICD, intracellular domain.

To predict the site of the interaction between σS-GVIIIA and mouse 5-HT_3_ receptor, AlphaFold 3 [38] was used. The predicted complex between σS-GVIIIA and the 5-HT_3_ receptor is shown in Figure 6. A comparison of this complex with the granisetron–receptor complex [41], a small molecule 5-HT_3_ receptor antagonist (Supplementary Figure S8), indicates similarity between the binding site of the small molecule antagonist and the peptide antagonist. The critical residues in the orthosteric binding site on the 5-HT_3_ receptor, established in previous studies with other ligands [41–45], are shown in silver stick format in Figure 6 and Supplementary Figure S8.

Analysis of the σS-GVIIIA and 5-HT_3_ receptor complex indicates that several peptide residues are within 4 Å of 5-HT_3_ receptor atoms (Figure 6). Inter-cysteine loop 7 of σS-GVIIIA (Figure 4), which includes the bromotryptophan at residue 34 (BTR34), is the main region predicted to interact with the active site of the 5-HT_3_ receptor. Tryptophan is a precursor to the endogenous agonist serotonin (5-hydroxytryptamine, 5-HT) and is thus structurally similar to serotonin. There are also additional regions of σS-GVIIIA, involving the β1 and β4 strands, which are predicted to interact outside of the active site. Several hydrogen bonds between the β-strands of the receptor and the β4 strand of σS-GVIIIA are predicted for this additional site of interaction. Residues 5–26 of σS-GVIIIA are not predicted to interact with the 5-HT_3_ receptor (Figure 6). In contrast with the mature GVIIIA sequence, the presence of the precursor sequence did not have a significant influence on the prediction results.

## Discussion

In this study, we have shown that σS-GVIIIA contains a GFCK structural motif. This represents the first example of a conotoxin, or indeed an invertebrate venom peptide, experimentally shown to have this structural motif. In conjunction with the earlier study on σS-GVIIIA [16], this is also the first example of a GFCK motif peptide being associated with 5-HT_3_ receptor antagonist activity.

Structural analysis of venom peptides is often carried out on synthetic/expressed versions of the peptides because of the limited availability of native material. Although the quantity of σS-GVIIIA purified from *C. geographus* venom was limited (0.6 mg), through the use of a 900 MHz NMR spectrometer with a cryogenically cooled probe, we were able to record high-quality NMR data, which allowed the determination of the 3D structure. Recording data on the native material ensured the native fold and native disulfide connectivity were present, and analysis of the sulfur–sulfur distances indicated that the disulfide connectivity corresponded to a GFCK motif (Figure 1, Supplementary Figure S4 and Table S1).

Additional disulfide bonds are often seen in GFCK-containing proteins (Supplementary Figure S6), including two additional inter-molecular disulfide bonds that facilitate functional dimer formation in the platelet-derived growth factor (PDGF) (PDB: 1PDG) [21,34]. σS-GVIIIA lacks such inter-molecular disulfide bonds, but forms two intra-molecular disulfide bonds, located in the region that is critical for receptor interaction based on our predicted ligand-receptor complex (Figure 6). Furthermore, the disulfide connectivities and topological locations of the two additional disulfide bonds present in σS-GVIIIA are generally not present in various signalling proteins and hormones [46,47] (Supplementary Figure S6). This difference in number of disulfide bonds may be critical for the receptor interaction [48]; thus, further study is warranted.

The prediction of protein/peptide structures by AI is a rapidly advancing area [49], and accurate structure prediction is critical to understanding the interactions and functions of proteins and peptides. We have shown that the presence/absence of the precursor sequence of σS-GVIIIA strongly influences the ability of AlphaFold 3 to correctly predict the mature σS-GVIIIA structure. The precursor sequence includes more conserved regions (e.g. signal sequence) than the mature sequence alone [37,50]; thus, this conservation may have influenced the AI structure prediction. We have recently identified similar limitations in AI-based prediction for the cysteine-rich peptide, conotoxin TxVIIB [51]. For new or rare classes of peptide/protein, reliable training data in AlphaFold 3 is currently lacking, and the outcome can be incorrect predictions of disulfide connectivities [51].

Based on the AlphaFold 3 prediction of the σS-GVIIIA-5–HT_3_ receptor complex, the solvent-exposed loop 7 region (residues 25–36) of σS-GVIIIA (Figure 4) was predicted to play a role in receptor binding (Figure 6). There is significant sequence variation among type VIII cysteine framework conotoxins in loop 7, along with both the loop 2 and 5 regions (Figure 4). Previous studies have indicated that σS-GVIIIA and σS-GVIIIB exhibit receptor specificity for the 5-HT_3_ receptor and the α9α10 subtype of nACh receptor, respectively [15,16]. The sequence variation in these loops is likely responsible for the differences in receptor selectivity observed for σS-GVIIIA and σS-GVIIIB against these therapeutic targets.

In the original study of σS-GVIIIA, bromotryptophan at position 34 was suggested to be a critical residue for the 5-HT_3_ receptor interaction based on the chemical similarity to the endogenous agonist serotonin [16]. In our study, the use of AI-based AlphaFold 3 structural prediction has supported this suggestion that bromotryptophan is located in a critical position to interact with the orthosteric binding site (Figure 5 and Supplementary Figure S8). Further experimental studies to confirm the detailed interaction of σS-GVIIIA with the 5-HT_3_ receptor are clearly warranted.

The 5-HT_3_ receptor has multiple subtypes [52], and the distribution of subtype expression appears to be associated with a range of clinical conditions and patient response to therapeutic drugs [19,53–55]. Analysis of the complex relationship in terms of binding of partial agonists, competitive, non-competitive antagonists or other allosteric modulators to this important therapeutic receptor has been revived in recent years [19,56,57]. The determination of the structure of the novel peptide antagonist σS-GVIIIA will further our understanding of the potential modes of interaction of peptides with 5-HT_3_ receptor subtypes, which in turn may facilitate the design of drug leads targeting this receptor.

Our observation of individual variability in the crude venom composition and the switch of the venom profile between defense- and predation-provoked stimuli was consistent with previous studies [58,59]. However, factors influencing the crude venom profile on each milking occasion, which had a major influence on the relative abundance of σS-GVIIIA in the defensive venom, warrant further investigation.

In conclusion, working with native venom material is challenging but offered us the opportunity to determine the native fold of σS-GVIIIA. Our study highlighted the variabilities in the crude venom composition of *C. geographus*, including differences between defensive and offensive venom. The NMR structure of σS-GVIIIA provided the first experimental evidence of the GFCK motif in conotoxins. Based on sequence similarity, it appears likely that several more known conotoxins also contain this GFCK motif. More experimental studies on conotoxins with the GFCK motif are likely to improve AI prediction of 3D structures of members of this structural family. As the prediction of σS-GVIIIA/5-HT_3_ receptor complex provided insight into further studies, the AI-based structural prediction of ligand–target interaction appears to be a promising approach to assist in designing/evaluating drug leads prior to synthesis. Our determination of the novel disulfide connectivity in the σS-GVIIIA conotoxin is also likely to facilitate the *in vitro* synthesis of this molecule through selective oxidation. Once this has been achieved, this will allow more detailed structure/function relationships to be explored experimentally and subsequent drug design studies for the therapeutically important 5-HT_3_ receptor.

## Materials and methods

### Venom sample milking

Cone snails (*C. geographus*) were collected from the Great Barrier Reef, Queensland, Australia, by a permitted local commercial supplier (Cairns Marine, Cairns, Australia) and kept alive in an aquarium laboratory facility at James Cook University (J. Seymour, eduQuarium). The cone snails were not killed during this project, but some died naturally. The venom was collected from individual animals by milking with defensive and offensive stimuli [58]. Briefly, a 1.5-ml tube covered with parafilm was prepared and the individual animal enticed via a threat (defensive) or prey item (offensive) to inject the venom into the tube through the parafilm. Milking was conducted over a 36-month period on approximately a once weekly basis. All venom samples were stored at −30°C until purification, pooled samples for each individual were generated by pooling all samples for each individual. To create the venom pools, each individual sample tube was centrifuged for 10 min at maximum speed in a benchtop centrifuge (MICRO 200 R, Hettichi, U.S.A., 15,000 rpm) and the supernatants for each specimen were pooled into one tube per specimen. The remaining sample precipitate was re-suspended with 0.5 ml of type 1 water and the same procedure repeated to collect further supernatant. The pooled venom sample was centrifuged one final time to ensure the removal of any larger particulates. Samples were not filtered to minimise sample loss. More than 20 fractions (~1.5 ml) were collected for each crude venom purification cycle. In total, two rounds of purification were conducted for each of the five pooled samples.

### Venom profile analysis

LC–MS analysis, on a Shimadzu LC-MS2020 single quadrupole mass spectrometer (Shimadzu, Kyoto, Japan) equipped with a Shimadzu Prominence high-performance liquid chromatography system, was used to identify the masses of each peptide present in the crude venom and generate a venom profile for each milking. The UV absorbance at 214 and 280 nm was used to monitor the relative abundance of σS-GVIIIA in each milking, which was observed to fluctuate during the pilot studies. The sample (2 µl in 8 µl LC-MS Buffer A) was loaded onto a RP-HPLC column (Phenomenex Aeris PEPTIDE, 150 mm × 2.1 mm, 3.6 µm, 100 Å; Phenomenex, Torrance, CA, USA) and eluted using a linear 1% gradient of LC–MS solvent B (90% ACN with 0.09% formic acid [FA] in type 1 water) in LC–MS solvent A (0.1% FA in type 1 water) at a flow rate of 250 µl/min. Electrospray ionisation mass spectrometry (ESI-MS) data were collected in positive ionisation mode over a scan range of 250–2000 m*/*z. Molecular weights were reconstructed from ion series using the ‘multi-charged ion analysis’ module of the Shimadzu LabSolutions software (Version 5.96) (Shimadzu, Kyoto, Japan).

### Isolation of σS-GVIIIA conotoxin from the crude venom with RP-HPLC

The pooled sample was diluted with HPLC solvent A (0.05% trifluoroacetic acid [TFA] in type 1 water) and purified using RP-HPLC (Agilent Infinity 1260: Agilent, Santa Clara, CA, U.S.A.). A total of 500 µl of the pooled sample was loaded onto a C18 semi-prep column (Phenomenex Aeris PEPTIDE XB-C18, 250 mm × 10 mm, 5 µm, 100 Å; Phenomenex, Torrance, CA, U.S.A.). Elution used a linear 1% gradient (0–60% solvent B over 60 min) of HPLC solvent B (90% acetonitrile (ACN) with 0.045% TFA in type 1 water) at a flow rate of 3 ml/min. Absorbance was monitored at 214 and 280 nm, and fractions were collected automatically into a 96-well, 2-ml deep well plate (Axygen, Union City, CA, U.S.A.) at 0.33 minute intervals.

An impure fraction containing σS-GVIIIA was re-purified using an analytical column (Phenomenex Aeris PEPTIDE XB-C18, 150 mm × 4.6 mm, 3.5 µm, 100 Å; Phenomenex, Torrance, CA, U.S.A.) with a linear gradient of 0.25% (30–50% HPLC solvent B over 80 min) at a flow rate of 1 ml/min and absorbance monitored at 214 nm and 280 nm. The purified σS-GVIIIA sample was lyophilised and stored at −30°C.

### Mass spectrometry

The mass of the peptide in the purified fraction (subsequently confirmed by NMR spectroscopy to be σS-GVIIIA conotoxin) was determined by mass spectrometry using a SCIEX TOF/TOF^™^ 5800 MALDI mass spectrometer (SCIEX, Framingham, MA, U.S.A.), mixing 0.75 µl of sample with 0.75 µl α-cyano-4-hydroxycinnamic acid (CHCA; Sigma-Aldrich, St. Louis, MO, U.S.A.) matrix at 7.5 mg/ml in 50% ACN/0.1% TFA onto an Opti-TOF 384-well plate. Data were collected in reflector positive ion mode with a scan range of 2000–5000 m/z and averaged over 2000 laser shots. An LC-MS2020 single quadrupole mass spectrometer (Shimadzu, Kyoto, Japan) was also used to confirm the mass and purity of the σS-GVIIIA sample. Sample (30 µl) was loaded onto a RP-HPLC column (Phenomenex Aeris PEPTIDE, 150 mm × 2.1 mm, 3.6 µm, 100 Å; Phenomenex, Torrance, CA, U.S.A.) and eluted using a linear 1% gradient of LC-MS Buffer B in LC-MS solvent A at a flow rate of 250 µL/min. ESI-MS data were collected in positive ionisation mode over a scan range of 250–2000 m*/*z. Molecular weights were reconstructed from ion series using the ‘multi-charged ion analysis’ module of the Shimadzu LabSolutions software (Version 5.96) (Shimadzu, Kyoto, Japan).

### NMR spectroscopy

Lyophilised sample was dissolved in 90% H_2_O:10% D_2_O at a concentration of approximately 0.26 mM. All NMR spectra were acquired on either a Bruker 600 MHz AVANCE III NMR spectrometer (Bruker, Karlsruhe, Germany) or Bruker Avance Neo 900 MHz spectrometer; both were equipped with cryogenically cooled probes. Two-dimensional spectra, including ^1^H-^1^H TOCSY, ^1^H-^1^H NOESY, ^1^H-^1^H DQF-COSY and ^1^H-^13^C HSQC, were collected at 298 K and recorded with a 1 s interscan delay using standard Bruker pulse sequences with an excitation sculpting scheme for solvent suppression. Homonuclear TOCSY and NOESY spectra were acquired with a mixing time of 80 ms and 200 ms, respectively. All spectra were processed using Bruker TopSpin (Version 4.3.0) and assigned using CcpNMR 2.4 (Collaborative Computing Project for NMR 2.4) analysis [60] based on the approach described in Wüthrich et al. [25,61].

### Structure calculations

The solution NMR structure of σS-GVIIIA was calculated using the CYANA program [26,27]. The experimental restraints, including distance, backbone torsion-angle and chi1 (χ1) angle, were incorporated into the calculation for the preliminary structure. The distance and angle restraints were derived from the NOESY spectra. Torsion-angle restraints were predicted using TALOS-N [30,31]. The side-chain χ1 angle for cysteine residues was determined based on the information from the intensities of interproton HN-Hβ and Hα–Hβ cross-peaks derived from NOESY spectra [62] and DISH (https://dish-s-407402.web.app/). DISH is a prediction algorithm for side-chain dihedral angles, such as *χ*1 and *χ*2 angles, based on the chemical shifts [63]. The *χ*1 angles from both sources agreed with each other and were used for the structure calculations. Hydrogen bond restraints were subsequently included based on experimental data from the temperature coefficients of the amide protons [64,65] and the preliminary structures. The most likely disulfide connectivities were predicted based on the distances between the sulfur atoms in the preliminary structures, measured using MOLMOL [32]. A set of 100 final structures was calculated with the most likely disulfide connectivity, and the 20 structures with the lowest target function chosen to present the final ensemble. Structures were visualized and the RMSD values were assessed using MOLMOL [32].

Disulfide bonds involving four cysteines (Cys^2^, Cys^17^, Cys^23^ and Cys^40^) were found to need further clarification based on the discrepancy between the AlphaFold prediction and the NMR structure. We calculated a set of structures for each of the three possible disulfide connectivity patterns possible with these four cysteine residues (Set 1, Set 2 and Set 3: Supplementary Figure S7). The coordinates and chemical shifts were deposited in the PDB (ID code 9EBE) and BMRB (ID code 31213), respectively.

### AlphaFold prediction of the 3D structure of σS-GVIIIA and σS-GVIIIA/5-HT_3_ complex

The neural network based AlphaFold is a novel computational approach for the prediction of peptide/protein structures, developed by Google Deep Mind. In this AI system, multi-sequence alignments, physical, biological and evolutionary knowledge are integrated into a sophisticated machine learning algorithm [66]. AlphaFold 3 became available in May 2024 on the AlphaFold Server [38].

We predicted the structure of σS-GVIIIA using AlphaFold 3 with or without a precursor sequence. This was based on a previous finding which indicated that the precursor sequences can significantly influence the success of AI structure predictions [67]. In the input sequence for AlphaFold 3, the hydroxyproline at residue 9 was able to be distinguished from the proline without a PTM at residue 31; however, there was no option in AlphaFold 3 to specify bromotryptophan at residue 34 or C-terminal amidation. The predicted results were visualised using MOLMOL [32].

Mouse 5-HT_3_ receptor was used for our AlphaFold prediction of the ligand-receptor complex between σS-GVIIIA and mouse 5-HT_3_ receptor [38], as multiple binding analyses of various antagonists have been studied with the mouse receptor, including the original σS-GVIIIA study [16]. The two known functional subtypes of the serotonin receptor are 5-HT_3A_ and 5-HT_3A/3B_; with the most studied functional subtype, 5-HT_3A_ being used for our analysis. The sequences of σS-GVIIIA with or without precursor were used for the prediction of the complex.

### Multiple sequence alignment of σS-GVIIIA with potentially structurally related proteins and peptides

The signature sequences of the GFCK motif (CXGXC and CXC) were manually searched against *Conus* venom peptides available in the ConoServer database [68] with the type-VIII framework to identify potential/unrecognised conotoxins containing the GFCK motif. Sequences were aligned based on the signature sequences and the distribution pattern of the other cysteine residues.

## Supplementary material

Online supplementary figures and tables

## Data Availability

The coordinates and chemical shifts were deposited in the PDB (rcsb.org: ID code 9EBE) and BMRB (bmrb.io: ID code 31213), respectively. All other data are contained within the manuscript. All data supporting the findings of this study are available from the corresponding author upon request.

## Abbreviations

GFCK: growth factor cystine knot
GPH-α: glycoprotein hormone α subunit
5-HT_3_ receptor: serotonin type 3 receptor
ICK: inhibitor cystine knot
LC–MS: liquid chromatography–mass spectrometry
NMR: nuclear magnetic resonance
RMSD: root-mean-square deviation
RP-HPLC/MS: reversed-phase high-performance liquid chromatography/mass spectrometry
σS-GVIIIA: sigma-S-GVIIIA
