# Supplementary material for: Structural characterisation of a cysteine-rich conotoxin, sigma(σ)S-GVIIIA, extracted from the defensive venom of the marine cone snail Conus geographus
Source: Biochem J. 2025 May 23;482(11):639–53. doi: 10.1042/BCJ20240753 (PMC12203953; doi:10.1042/BCJ20240753)
Supplement: Online supplementary figures and tables [file BCJ-482-11-BCJ20240753-s001.docx]

**Structural characterisation of a cysteine-rich conotoxin, sigma(σ)S-GVIIIA, extracted from the defensive venom of the marine cone snail *Conus geographus***

Yoshimi Peck^1^, David T. Wilson^1^, Danica Lennox-Bulow^1^, Julien Giribaldi^2^, Jamie Seymour^1^, Sebastien Dutertre^2^, K. Johan Rosengren^3^, Michael J. Liddell^4^, Norelle L. Daly^1^

^1^Australian Institute of Tropical Health and Medicine, James Cook University, Cairns, QLD, Australia. ^2^IBMM, University of Montpellier, CNRS, ENSCM, Montpellier, France. ^3^School of Biomedical Sciences, The University of Queensland, Brisbane, QLD, Australia. ^4^College of Science and Engineering, James Cook University, Cairns, QLD, Australia.

**Correspondence**: Norelle Daly ([norelle.daly@jcu.edu.au](mailto:norelle.daly@jcu.edu.au))

**Supplementary Table S1: The mean, standard deviation (SD), and minimum and maximum distances between sulfur atoms of each cysteine, sorted by the shortest mean sulfur-sulfur (S-S) distance.**

| Cysteine pair | | | S-S distance | | | | |
| --- | --- | --- | --- | --- | --- | --- | --- |
|  |  |  | Mean±SD (Å) | | | Minimum (Å) | Maximum (Å) |
| **15** | **-** | **38** | **3.0** | **±** | **1.0** | **0.8** | **4.8** |
| **6** | **-** | **25** | **3.6** | **±** | **1.3** | **2.3** | **6.0** |
| **11** | **-** | **36** | **3.8** | **±** | **1.7** | **1.2** | **7.9** |
| **2** | **-** | **17** | **3.9** | **±** | **1.0** | **1.8** | **5.4** |
| **23** | **-** | **40** | **4.1** | **±** | **1.4** | **1.6** | **5.6** |
| 15 | - | 25 | 4.5 | ± | 0.7 | 3.7 | 6.4 |
| 17 | - | 23 | 4.7 | ± | 0.8 | 3.4 | 5.7 |
| 25 | - | 38 | 5.5 | ± | 1.0 | 3.7 | 7.1 |
| 6 | - | 15 | 5.8 | ± | 0.8 | 4.9 | 7.4 |
| 25 | - | 36 | 5.8 | ± | 1.2 | 4.1 | 8.7 |
| 23 | - | 38 | 6.3 | ± | 0.8 | 5.0 | 7.9 |
| 17 | - | 38 | 6.4 | ± | 1.4 | 4.2 | 9.4 |
| 11 | - | 25 | 6.5 | ± | 1.5 | 3.3 | 8.3 |
| 17 | - | 40 | 6.6 | ± | 1.5 | 3.8 | 9.0 |
| 6 | - | 36 | 6.7 | ± | 2.2 | 3.1 | 11.9 |
| 15 | - | 23 | 6.8 | ± | 0.7 | 4.9 | 7.6 |
| 6 | - | 38 | 7.3 | ± | 1.5 | 4.6 | 10.7 |
| 38 | - | 40 | 7.4 | ± | 1.2 | 4.7 | 9.2 |
| 6 | - | 11 | 7.5 | ± | 1.4 | 5.7 | 11.0 |
| 15 | - | 17 | 7.6 | ± | 1.5 | 4.0 | 9.8 |
| 2 | - | 23 | 7.9 | ± | 0.9 | 6.6 | 9.4 |
| 2 | - | 38 | 8.3 | ± | 1.4 | 6.6 | 11.1 |
| 15 | - | 40 | 8.9 | ± | 1.7 | 4.9 | 11.2 |
| 2 | - | 15 | 9.2 | ± | 1.2 | 6.2 | 10.9 |
| 36 | - | 38 | 9.7 | ± | 0.9 | 8.4 | 11.6 |
| 15 | - | 36 | 9.8 | ± | 1.4 | 7.4 | 13.0 |
| 2 | - | 40 | 10.0 | ± | 1.9 | 6.9 | 13.3 |
| 23 | - | 25 | 10.4 | ± | 0.3 | 10.0 | 11.4 |
| 11 | - | 15 | 10.9 | ± | 1.5 | 7.8 | 12.5 |
| 11 | - | 38 | 11.4 | ± | 2.0 | 6.6 | 13.8 |
| 25 | - | 40 | 11.5 | ± | 1.2 | 9.4 | 12.9 |
| 17 | - | 25 | 11.6 | ± | 1.3 | 10.0 | 13.3 |
| 6 | - | 23 | 12.4 | ± | 0.7 | 11.2 | 13.9 |
| 6 | - | 17 | 12.8 | ± | 1.4 | 11.2 | 15.5 |
| 2 | - | 25 | 13.2 | ± | 1.0 | 11.6 | 15.2 |
| 2 | - | 6 | 13.5 | ± | 1.0 | 11.5 | 15.4 |
| 6 | - | 40 | 14.0 | ± | 1.6 | 11.0 | 17.1 |
| 23 | - | 36 | 15.3 | ± | 1.1 | 13.5 | 18.1 |
| 36 | - | 40 | 15.4 | ± | 0.8 | 13.9 | 16.1 |
| 17 | - | 36 | 15.9 | ± | 1.5 | 14.0 | 18.6 |
| 11 | - | 23 | 16.4 | ± | 1.4 | 13.5 | 18.3 |
| 11 | - | 40 | 16.7 | ± | 1.8 | 12.1 | 18.9 |
| 2 | - | 36 | 17.1 | ± | 1.7 | 14.8 | 21.5 |
| 11 | - | 17 | 17.6 | ± | 2.0 | 13.6 | 20.9 |
| 2 | - | 11 | 19.0 | ± | 2.0 | 16.4 | 22.9 |

**Supplementary figure legends**

**Supplementary Figure S1. A comparison of the LC-MS chromatograms of crude defensive venom of *C. geographus* extracted from milkings of different specimens, on different days, or different stimuli. A.** Specimen A on Day W. **B.** Specimen B on Day W. **C.** Specimen B on Day X. σS-GVIIIA is highlighted in red. **D.** **Above.** Specimen C on Day Y with defense-evoked stimuli. **Below**. Specimen C on Day Z with predation-evoked stimuli. A-C show UV data (214 nm) and Total Ion Current (TIC) is shown in D.

**Supplementary Figure S2. The RP-HPLC chromatogram of pooled crude venom of *C. geographus.*** σS-GVIIIA is highlighted in magenta.

**Supplementary Figure S3. The mass spectrum of purified fraction of σS-GVIIIA.** The results of an ion-series reconstruction of the three ions in the mass spectrum is shown in the table below the spectrum.

**Supplementary Figure S4. Analysis of the sulfur-sulfur distances in σS-GVIIIA.** Sulfur-sulfur (S-S) distances between pairs of cysteine residues in each of the 20 structures of σS-GVIIIA calculated without disulfide bond restraints are shown. When the distance is <3.5 Å the bars are shown in green, between 3.5 Å and 6 Å in blue and more than 6 Å in red. Distances above 20 Å are shown as 20 Å. The mean, minimum and maximum distance of each group are summarised in supplementary Table 1.

**Supplementary Figure S5. The structure ensemble, containing the twenty structures with the lowest target function, of the final solution NMR structure of σS-GVIIIA (PDB:9EBE).** The five disulfide bonds are shown in navy.

**Supplementary Figure S6. A comparison of additional disulfide bonds in GFCK containing proteins.** The 3D structure and sequence alignment of selected examples of GFCK containing proteins are shown. GPH-α: Glycoprotein hormone α subunit. GPH-α2: Glycoprotein hormone α2 subunit. Thyro-α: Thyrostimulin α subunit. VEGF-B: Vascular endothelial growth factor B. PDGF-B: Platelet-Derived Growth Factor B.

**Supplementary Figure S7.**

**The comparison of the 3D structure, target function and restraint violations among the three sets of disulfide connectivities for the structure of σS-GVIIIA . A. Cys^2^-Cys^17^ and Cys^23^-Cys^40^. B. Cys^2^-Cys^23^ and Cys^17^-Cys^40^. C. Cys^2^-Cys^40^ and Cys^17^-Cys^23^.** The structure with the lowest target function is shown. The schematic figures of each set highlight the difference in the disulfide connectivities amongst the three sets.

**Supplementary Figure S8. Comparison of the binding of granisetron and σS-GVIIIA at the orthosteric binding site of the 5-HT_3_ receptor.** The key residues on the orthosteric binding site on the 5-HT_3_ receptor are shown in silver stick format. Granisetron is shown in blue. The σS-GVIIIA conotoxin is shown in magenta. Bromotryptophan at residue 34 (BTR34) of σS-GVIIIA is shown in magenta stick format. The σS-GVIIIA consists of four β-strands, which are labelled as β1 to β4.

Supplementary Figure S1


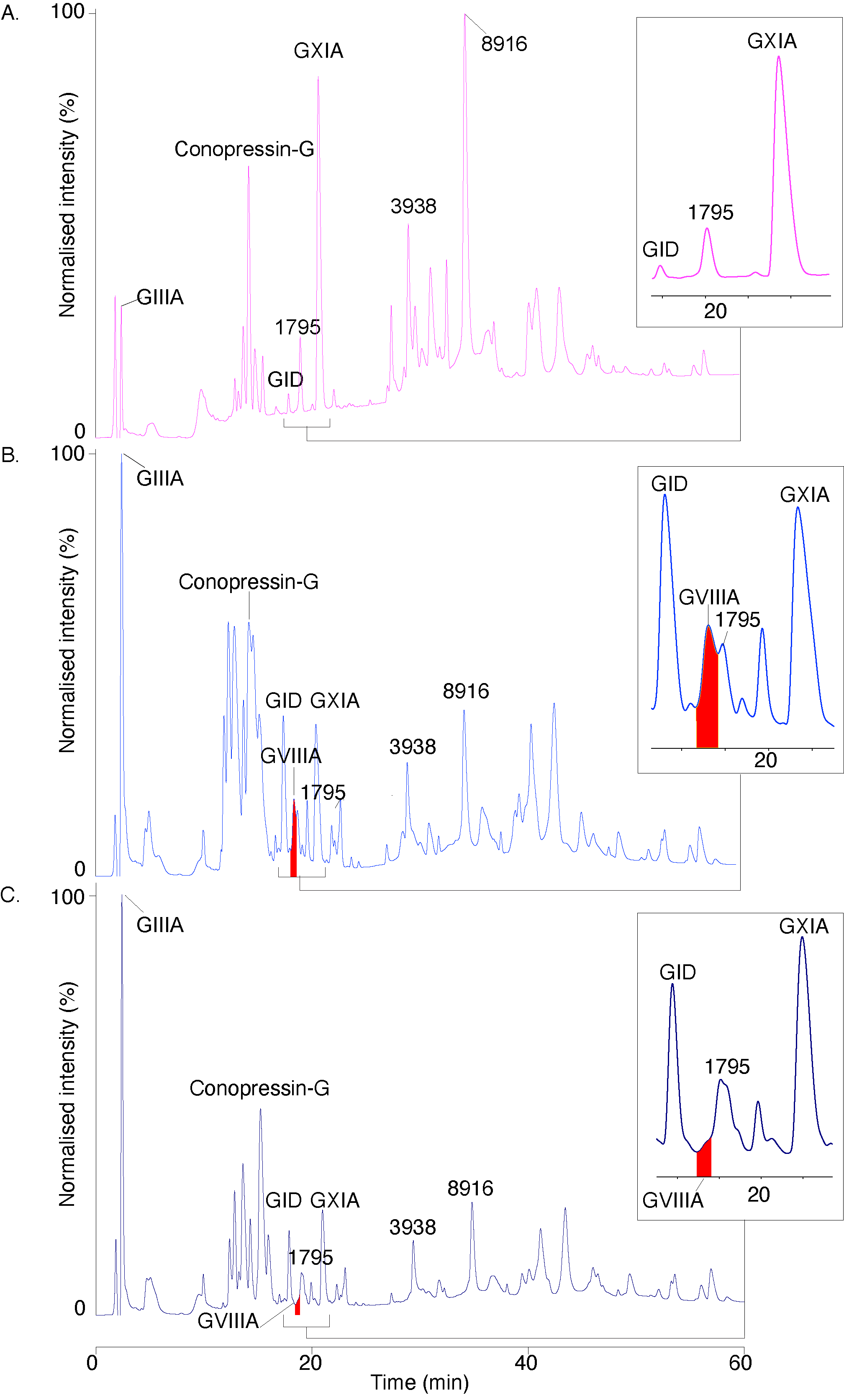





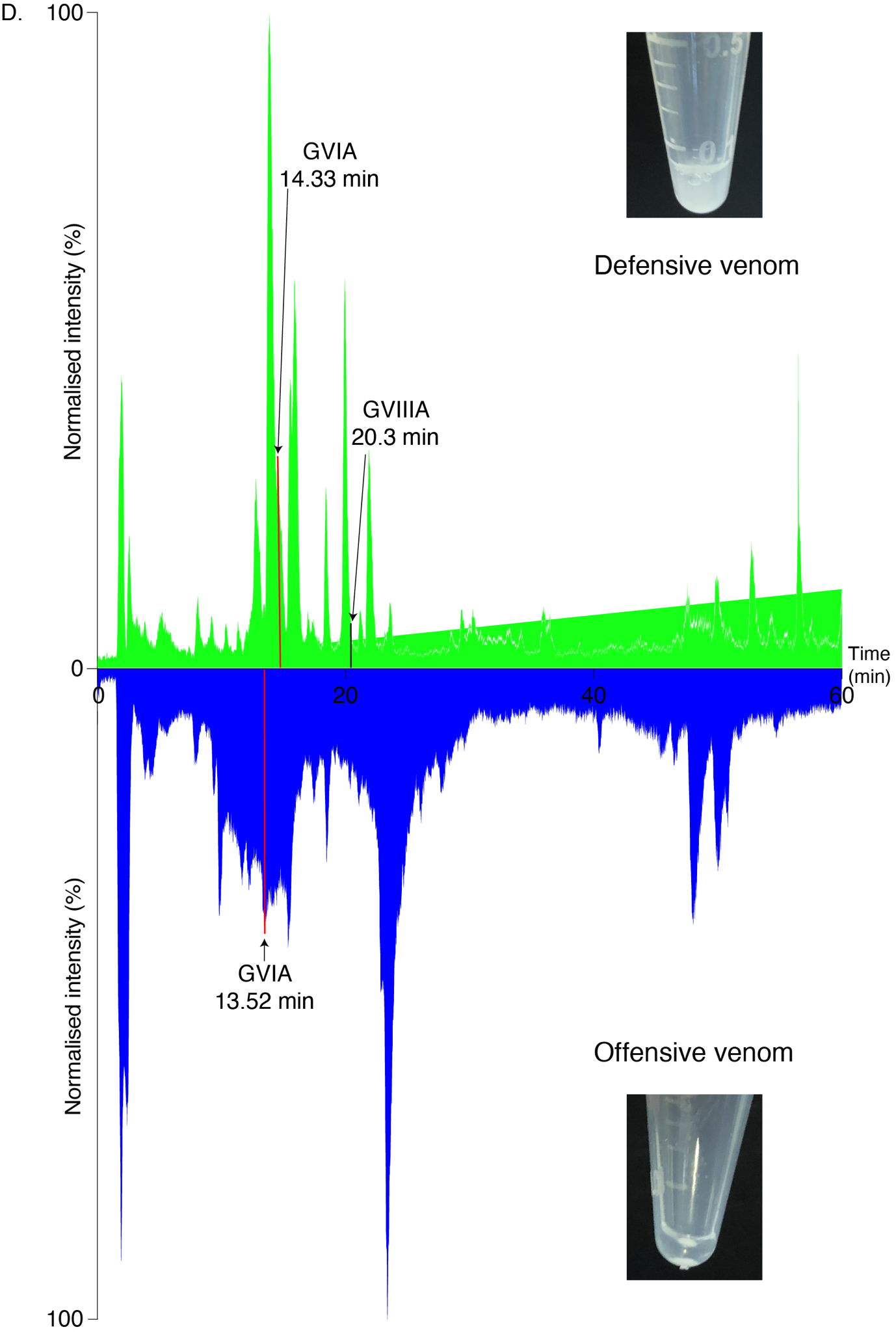


Supplementary Figure S2


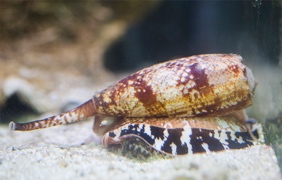

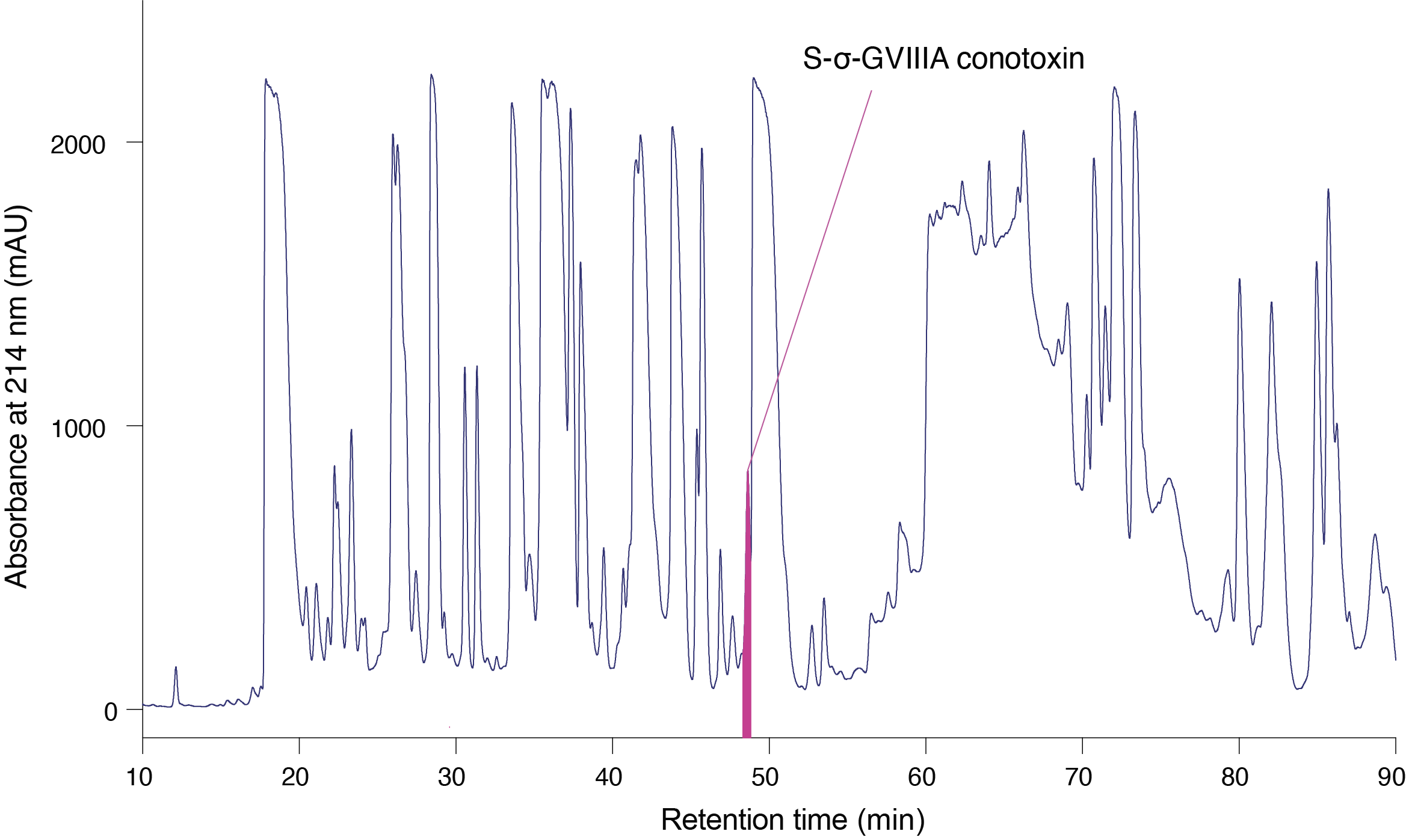


Supplementary Figure S3


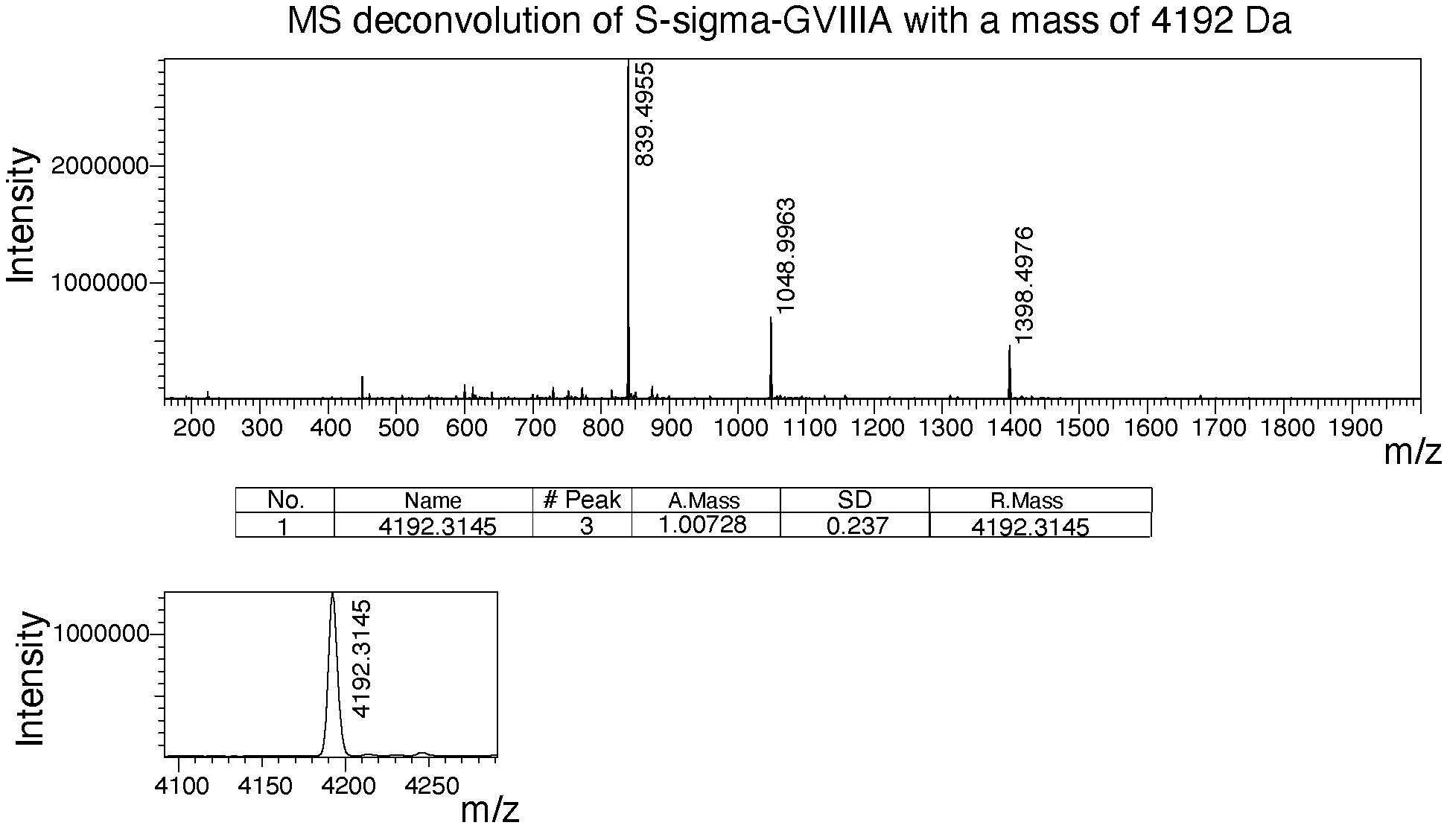


Supplementary Figure S4


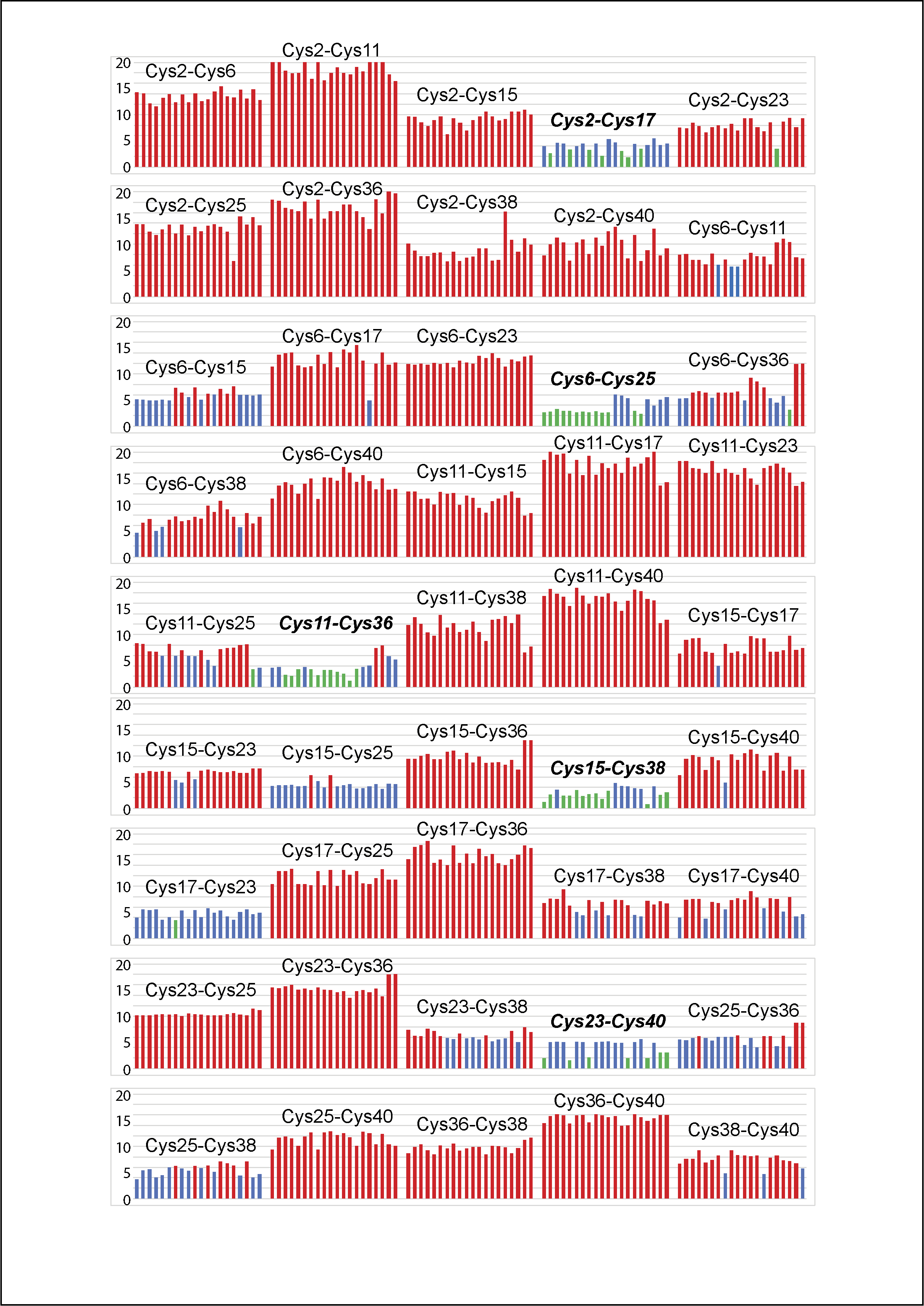


Supplementary Figure S5


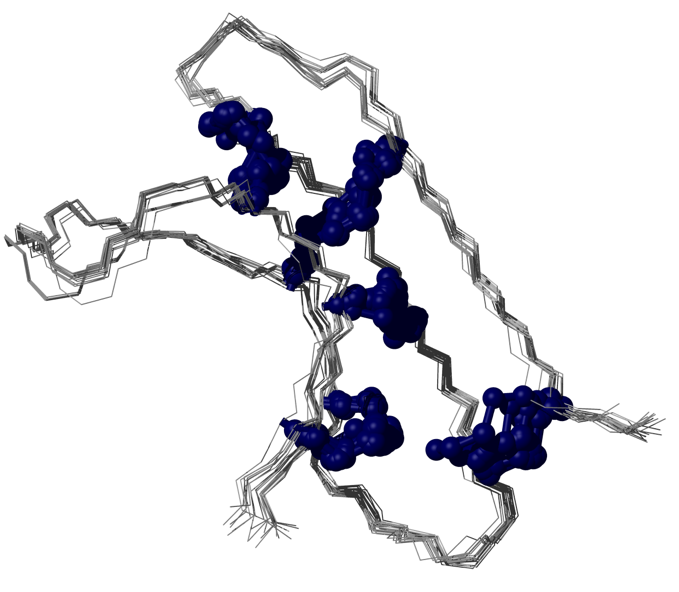


Supplementary Figure S6

Supplementary Figure S7

Supplementary Figure S8
